# Prevalence, Genetic Diversity, and Zoonotic Transmission Potential of *Cryptosporidium* spp. in Snakes: A Systematic Review and Meta‐Analysis

**DOI:** 10.1002/vms3.71167

**Published:** 2026-08-12

**Authors:** Ali Asghari, Ali Pouryousef, Kambiz Karimi, Amin Kheiri, Mohammad Reza Soltani, Farajolah Maleki

**Affiliations:** ^1^ Department of Basic Medical Sciences Khoy University of Medical Sciences Khoy Iran; ^2^ Leishmaniasis Research Center Sabzevar University of Medical Sciences Sabzevar Iran; ^3^ Department of Medical Parasitology and Mycology School of Medicine, Shiraz University of Medical Sciences Shiraz Iran; ^4^ Zoonotic Diseases Research Center Ilam University of Medical Sciences Ilam Iran; ^5^ Department of Endodontics Faculty of Dentistry Ilam University of Medical Sciences Ilam Iran; ^6^ Department of Operative Dentistry School of Dentistry Ilam University of Medical of Sciences Ilam Iran; ^7^ Clinical Research Development Unit Shahid Mostafa Khomeini Hospital Ilam University of Medical Sciences Ilam Iran

**Keywords:** *Cryptosporidium* spp, cryptosporidiosis, reptile, snakes, systematic review, zoonotic transmission

## Abstract

**Background:**

*Cryptosporidium* spp. are important protozoan parasites infecting a wide range of vertebrate hosts, including reptiles. In snakes, this parasite is primarily associated with chronic gastrointestinal disease and substantial genetic diversity, including potentially zoonotic species. However, epidemiological data remain fragmented and heterogeneous. Therefore, this systematic review and meta‐analysis aimed to evaluate the prevalence, genetic diversity, and zoonotic potential of *Cryptosporidium* spp. in snakes.

**Methods:**

A systematic search was conducted in PubMed, Scopus and Web of Science from database inception to 5 May 2026. Additional studies were identified through Google Scholar and reference screening. Eligible studies reporting *Cryptosporidium* spp. prevalence in snakes were included. Pooled prevalence estimates and 95% confidence intervals (CIs) were calculated using a random‐effects model. Subgroup analyses, sensitivity analysis, meta‐regression and publication bias assessment were also performed.

**Results:**

A total of 35 studies comprising 36 datasets were included. The pooled prevalence of *Cryptosporidium* spp. in snakes was 12.6% (95% CI: 9.2%–17.0%), with substantial heterogeneity (*I* ^2^ = 92.1%). Captive‐associated snakes showed higher pooled prevalence (12.7%) than wild/free‐ranging snakes (8.7%). Molecular methods yielded higher prevalence estimates than microscopic techniques. Sensitivity analysis demonstrated stable pooled estimates (11.7%–13.6%). Meta‐regression showed a significant association between sample size and prevalence, whereas publication year was not significant. Egger's test indicated significant publication bias (*p* = 0.02). Eight *Cryptosporidium* species were identified in snakes, including *Cryptosporidium serpentis*, *Cryptosporidium tyzzeri*, *Cryptosporidium varanii*, *Cryptosporidium muris*, *Cryptosporidium parvum*, *Cryptosporidium baileyi*, *Cryptosporidium andersoni* and *Cryptosporidium hominis*. The most frequently reported species were *C. serpentis*, *C. tyzzeri*, *C. varanii*, and *C. muris*. Several zoonotic species, including *C. muris*, *C. parvum*, *C. tyzzeri*, *C. baileyi*, *C. andersoni* and *C. hominis* were detected mostly in captive‐associated snakes.

**Conclusions:**

The present study demonstrates that *Cryptosporidium* infection is relatively common in snakes and exhibits considerable genetic diversity. The detection of zoonotic species highlights the potential epidemiological importance of snakes within a One Health context, particularly in captive‐associated environments. However, substantial heterogeneity, limited data from wild populations and incomplete molecular characterization warrant cautious interpretation of the findings. Further large‐scale molecular epidemiological studies are needed to better clarify the ecology and zoonotic significance of *Cryptosporidium* spp. in snakes.

## Graphical abstract

This study provides the first global systematic synthesis of *Cryptosporidium* spp. infection in snakes. A pooled prevalence of 12.6% was identified, with higher occurrence in captive‐associated populations than in wild snakes. Eight *Cryptosporidium* species, including multiple zoonotic taxa, were reported, underscoring the potential role of snakes within the One Health framework. These findings establish an important baseline for future epidemiological surveillance, molecular characterization and risk assessment of reptile‐associated cryptosporidiosis.

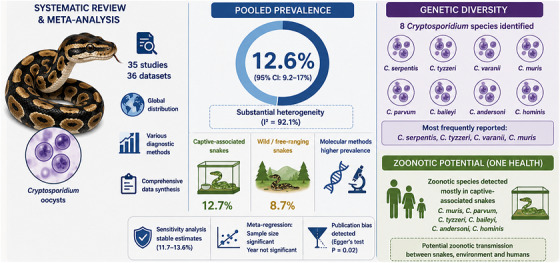

## 1 Introduction

*Cryptosporidium* is a globally distributed protozoan parasite belonging to the phylum Apicomplexa that infects a broad range of vertebrate hosts, including mammals, birds, reptiles, amphibians and fish (Putignani and Menichella 2010; Helmy and Hafez 2022). Members of the genus colonize epithelial cells of the gastrointestinal tract and are responsible for cryptosporidiosis, an important enteric disease of both veterinary and public health significance (Certad et al. 2017). Transmission occurs primarily through the faecal–oral route via environmentally resistant oocysts that can contaminate water, food, fomites and animal enclosures (Pumipuntu and Piratae 2018). Due to the low infectious dose, prolonged environmental persistence of oocysts, and resistance to many conventional disinfectants, *Cryptosporidium* spp. are considered among the most successful waterborne and foodborne parasitic pathogens worldwide (Adeyemo et al. 2019).

Over the past two decades, substantial advances in molecular epidemiology and phylogenetics have considerably expanded the taxonomy of *Cryptosporidium* spp. (Tipu et al. 2024). More than 40 valid species and numerous host‐adapted genotypes have now been recognized, many of which exhibit varying degrees of host specificity and zoonotic potential (Ryan et al. 2021). Among humans, the most clinically important species are *Cryptosporidium hominis* and *Cryptosporidium parvum*, although other species including *Cryptosporidium meleagridis*, *Cryptosporidium canis*, *Cryptosporidium felis*, *Cryptosporidium ubiquitum*, *Cryptosporidium cuniculus*, *Cryptosporidium viatorum*, *Cryptosporidium muris*, *Cryptosporidium baileyi*, *Cryptosporidium andersoni*, *Cryptosporidium erinacei*, *Cryptosporidium tyzzeri*, *Cryptosporidium bovis*, *Cryptosporidium suis*, *Cryptosporidium scrofarum*, *Cryptosporidium occultus*, *Cryptosporidium xiaoi*, *Cryptosporidium fayeri*, *Cryptosporidium ditrichi* and *Cryptosporidium mortiferum* have also been implicated in human infections (Ryan et al. 2021, Shi et al. 2025). Molecular tools targeting loci such as the small subunit ribosomal RNA (SSU rRNA), gp60, actin, and HSP70 genes have significantly improved species identification, subtype characterization, and understanding of transmission dynamics across host populations (Xu et al. 2020, Mahmudunnabi et al. 2024).

Reptiles, particularly snakes, are considered highly susceptible to cryptosporidiosis, and infections in these hosts are often chronic, progressive, and clinically significant (Louro et al. 2025). Historically, *Cryptosporidium serpentis* has been regarded as the principal pathogenic species infecting snakes, primarily colonizing the gastric epithelium and causing hypertrophic gastritis, regurgitation, anorexia, lethargy, progressive emaciation and eventual death (Louro et al. 2025, Y. Zhang et al. 2024). In contrast, *Cryptosporidium varanii* (formerly *Cryptosporidium saurophilum*) is more commonly associated with intestinal infections in lizards but has occasionally been detected in snakes (L. Xiao et al. 2004, Richter et al. 2011).

In recent years, the growing global trade and captive maintenance of reptiles as pets, zoo exhibits, breeding stock and research animals have increased concerns regarding the epidemiology and transmission dynamics of reptilian cryptosporidiosis. Captive‐associated conditions, including high animal density, chronic stress, shared husbandry equipment and prolonged environmental contamination, may facilitate parasite persistence and transmission among snakes (Garcia‐R et al. 2023, Mendoza‐Roldan et al. 2020). Despite increasing molecular evidence of *Cryptosporidium* spp. diversity in reptiles, available studies remain geographically fragmented, methodologically heterogeneous, and often limited by small sample sizes or inconsistent reporting of host management conditions. Moreover, the prevalence, molecular diversity, and zoonotic implications of *Cryptosporidium* spp. infections in snakes have not yet been comprehensively synthesized at the global level.

Therefore, the present systematic review and meta‐analysis aimed to comprehensively evaluate the global prevalence, genetic diversity, and zoonotic transmission potential of *Cryptosporidium* spp. in snakes.

## 2 Methods

### 2.1 Study Design and Reporting Framework

The present study was designed as a systematic review and meta‐analysis to comprehensively evaluate the global prevalence, genetic diversity, and zoonotic transmission potential of *Cryptosporidium* spp. in snakes. The design, implementation, and reporting of this study were performed in accordance with the Preferred Reporting Items for Systematic Reviews and Meta‐Analyses (PRISMA) guidelines (Moher et al. 2015, Page et al. 2021). The PRISMA 2020 checklist used for reporting this systematic review is provided in Table S1.

### 2.2 Search Strategy

A comprehensive literature search was conducted in three international electronic databases, including PubMed, Web of Science and Scopus, without time restriction up to 5 May 5 2026. The search strategy was developed using combinations of Medical Subject Headings (MeSH) terms, free‐text keywords and Boolean operators (′AND' and ′OR′) to maximize retrieval sensitivity. The main search terms included combinations of the following keywords: ′*Cryptosporidium*′, ′cryptosporidiosis', ′snake', ′reptile', ′prevalence', ′genetic diversity', ′genotype', ′species' and ′zoonotic transmission′. To minimize the possibility of missing relevant publications, the first 100 pages of Google Scholar were manually screened for potentially eligible studies not indexed in the primary databases. Additionally, the reference lists of all included articles and relevant review papers were manually examined to identify further eligible studies.

### 2.3 Eligibility Criteria

Studies were selected according to predefined inclusion and exclusion criteria. Studies were considered eligible if they met the following criteria: investigated *Cryptosporidium* spp. in snakes, reported prevalence data or provided sufficient information to calculate prevalence, used at least one diagnostic method for detecting *Cryptosporidium* spp. (including microscopic, immunological or molecular techniques), included original research data, reported sample size and number of positive cases, were published in peer‐reviewed journals, were published in English or had an English abstract containing extractable data.

Studies were excluded if they were review articles, editorials, letters or case reports without extractable prevalence data; did not specifically investigate snakes; lacked sufficient epidemiological information; reported only experimental infections without natural prevalence data; used duplicated datasets already included in another publication; reported enclosure‐level positivity without individual‐level infection data and had inaccessible full texts after reasonable attempts to obtain them.

### 2.4 Data Extraction

Data extraction was independently performed by two reviewers using a standardized data collection form. Prior to data extraction, the same two reviewers independently screened all retrieved records by title and abstract, followed by full‐text assessment of potentially eligible studies according to the predefined inclusion and exclusion criteria. Any disagreements regarding study eligibility or extracted data were resolved through discussion, and when consensus could not be reached, a third reviewer was consulted to achieve a final decision.

The following variables were extracted from each eligible study: first author's name, publication year, sampling year (when available), country and continent, snake management status (captive‐associated or wild/free‐ranging), total sample size, number of positive samples, reported prevalence of *Cryptosporidium* spp., detected *Cryptosporidium* species and diagnostic method used (microscopic or molecular).

For studies using multiple diagnostic approaches, molecularly confirmed prevalence data were preferentially extracted for quantitative synthesis because of their higher diagnostic specificity. When ambiguities were encountered, the original text, tables, supplementary materials and methodological descriptions were carefully reviewed to ensure accurate and consistent data extraction.

Snake populations maintained under human‐controlled conditions, including pets, zoological collections, breeding facilities, farms, sanctuaries, rescue centres and private collections, were categorized as captive‐associated populations. Snakes sampled directly from natural habitats were classified as wild/free‐ranging populations.

### 2.5 Quality Assessment

Methodological quality assessment of the included studies was performed using the Joanna Briggs Institute (JBI) Critical Appraisal Checklist for Prevalence Studies. The evaluation was based on eight methodological questions included in the checklist. Each item was scored as follows: ′Yes′ = 1 point, ′Unclear′ = 0.5 point and ′No′ = 0 point. The total quality score for each study was calculated by summing individual item scores. Based on the final score, studies were categorized as follows: low quality: < 3.5, moderate quality: 4–6.5 and high quality: > 6.5. The quality assessment results were used solely to evaluate the methodological rigor of the included studies and were not incorporated into the statistical meta‐analysis models.

### 2.6 Statistical Analysis

All statistical analyses were performed using Comprehensive Meta‐Analysis (CMA) software version 3 (Shamsi et al. 2025). The pooled prevalence of *Cryptosporidium* spp. in snakes was estimated using a random‐effects model with 95% confidence intervals (CIs), considering the expected heterogeneity among studies conducted across different geographical regions, snake populations and diagnostic methodologies. Heterogeneity among studies was evaluated using Cochran's *Q* statistic and the *I* ^2^ index (Mamizadeh et al. 2025). An I^2^ value greater than 75% was considered indicative of high heterogeneity. Subgroup analyses were conducted to investigate potential sources of heterogeneity according to: publication year, country, continent, sample size (≤ 100 vs. > 100), diagnostic method (microscopic vs. molecular) and snake management status (captive‐associated vs. wild/free‐ranging). Sensitivity analysis was performed using a leave‐one‐out approach in which each study was sequentially removed to evaluate its influence on the overall pooled prevalence estimate. Univariable meta‐regression analyses were conducted to assess the potential effects of publication year and sample size on prevalence estimates. Publication bias was evaluated visually using funnel plots and statistically using Egger's regression asymmetry test. A *p*‐value less than 0.05 was considered statistically significant for all analyses.

## 3 Results

### 3.1 Study Selection

**FIGURE 1:**
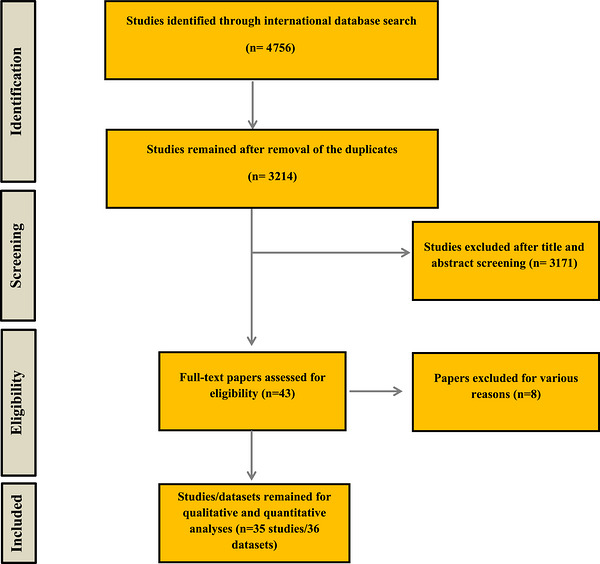
Flowchart depicting the process of included studies in this systematic review.

A total of 4756 records were identified through systematic searches of PubMed, Scopus and Web of Science databases. Additional potentially relevant studies were identified through manual screening of Google Scholar and reference lists of eligible articles. After removal of duplicate records, 3214 articles remained for title and abstract screening. Following initial screening, 43 studies were selected for full‐text evaluation. Among these, eight studies were excluded for various reasons, including lack of extractable prevalence data (Helmy and Hafez 2022), absence of snake‐specific results (Certad et al. 2017), experimental infection design (Putignani and Menichella 2010), duplicated datasets (Putignani and Menichella 2010) and enclosure‐level reporting without individual‐level data (Putignani and Menichella 2010). Ultimately, 35 studies comprising 36 independent datasets were included in the present systematic review and meta‐analysis (Louro et al. 2025, Y. Zhang et al. 2024, da Silva et al. 2014, Graczyk and Cranfield 2000, Hallinger et al. 2020, Hellebuyck et al. 2007, Karasawa et al. 2002, Karim et al. 2014, Kuroki et al. 2008, F. Li et al. 2025, Lobão et al. 2023, Mendoza‐Roldan et al. 2024, Richter et al. 2011, O'Hanlon et al. 2023, Papini et al. 2011, Pedraza‐Díaz et al. 2009, Polláková et al. 2025, Rataj et al. 2011, Richter et al. 2008, Rinaldi et al. 2012, Ruggiero et al. 2011, Satour and Dewir 2018, Sutthikornchai et al. 2026, Abe and Matsubara 2015, X. Xiao et al. 2019, Yimming et al. 2016, Žahourek and Kvičerová 2025, H. Zhang et al. 2020, Ziegler et al. 2007, Akhila et al. 2018, Alves et al. 2005, Diaz Fernandez et al. 2011, Beck 2025, Chai et al. 2021, Dang et al. 2008). One study contributed two independent datasets because it separately investigated captive‐associated and wild/free‐ranging snake populations. The detailed study selection process is presented in the PRISMA flow diagram (Figure 1).

### 3.2 Characteristics of Included Studies and Quality Assessment

The studies included in this meta‐analysis were published between 2000 and 2026 and represented snake populations from multiple geographic regions, including Africa, Asia, Europe, North America and South America. A total of 35 studies (36 datasets) involving both captive‐associated and wild/free‐ranging snake populations were analysed. Sample sizes varied considerably among studies, ranging from 2–750 snakes. Most studies were conducted in captive‐associated snake populations, including pet snakes, zoo‐kept snakes, breeding collections, sanctuaries, farms and private collections, whereas only four datasets investigated wild/free‐ranging snake populations. Molecular diagnostic approaches were more frequently used than microscopic methods (Table 1). Quality assessment based on the JBI checklist demonstrated that the majority of included studies were of moderate methodological quality, while several studies were categorized as high quality (Table S2).

**TABLE 1:** The main characteristics of the 35 studies/36 datasets included in the present study on the prevalence and genetic diversity of *Cryptosporidium* spp. in snakes.

Author, year	Sampling time	Country	Snake type	Total no.	Infected no.	Prevalence (%)	Detection method	*Cryptosporidium* species reported (*n*)
Graczyk and Cranfield, 2000	UC	USA	CA	90	13	14.4	MIC	*C. serpentis* (13)
Karasawa, 2002	2000–2001	Brazil	CA	50	7	14	MIC	*Cryptosporidium* spp. (7)
Alves, 2005	2002–2003	Portugal	CA	16	0	0	MOL	—
Ziegler, 2007	1996–1998	USA	W/F	2	0	0	MOL	—
Hellebuyck, 2007	UC	Belgium	CA	105	0	0	MIC	—
Dang HaiLiang, 2008	UC	China	CA	31	14	45.2	MIC	*Cryptosporidium* spp. (14)
Kuroki, 2008	2001–2007	Japan	CA	469	57	12.1	MOL	*Cryptosporidium* spp. (57)
Richter, 2008	1996–2005	Austria	CA	182	7	3.8	MOL	*Cryptosporidium* spp. (7)
Pedraza‐Diaz, 2009	UC	Spain	CA	65	30	46.1	MOL	*C. varanii* (1), *C. serpentis* (24), *C. muris* (2), *C. tyzzeri* (1), *Cryptosporidium* spp. (2)
Papini, 2011	2009–2010	Italy	CA	74	0	0	MIC	—
Rataj, 2011	2000–2005	Slovenia*	CA	55	1	1.8	MIC	*Cryptosporidium* spp. (1)
Diaz Fernandez, 2011	UC	Italy	CA	21	14	66.7	MOL	*C. tyzzeri* (10), *C. muris* (3), *Cryptosporidium* spp. (1)
Ruggiero, 2011	UC	Brazil	CA	25	11	44	MOL	*C. serpentis* (11)
Richter, 2011	UC	Austria	CA	174	39	22.4	MOL	*C. muris* (2), *Cryptosporidium* spp.(16), *C. baileyi* (2), *C. varanii* (19)
Rinaldi, 2012	2009–2010	Italy	CA	125	6	4.8	MOL	*C. serpentis* (3), *Cryptosporidium* spp. (3)
Da Silvaa, 2014	UC	Brazil	CA	503	60	11.9	MOL	*C. serpentis* (15), *C. varanii* (12), *C. tyzzeri* (7), *C. muris* (4), *Cryptosporidium* spp. (22)
Karim, 2014	2012	China	CA	240	3	1.3	MOL	*Cryptosporidium* spp. (3)
Abe and Matsubara, 2015	2012–2014	Japan	CA	2	2	100	MOL	*C. serpentis* (1), *C. varanii* (1)
Yimming, 2016	UC	Thailand	CA	165	40	24.2	MOL	*C. parvum* (24), *C. tyzzeri* (2), *C. serpentis* (9), *C. varanii* (1), *C. andersoni* (1), *C. muris* (3)
Satour, 2018	2017–2018	Egypt	CA	26	6	23	MIC	*Cryptosporidium* spp. (6)
Akhila, 2018	2016–2017	India	CA	49	7	14.3	MIC	*Cryptosporidium* spp. (7)
Xiao, 2019	2018	China	CA	149	15	10.1	MOL	*C. baileyi* (10), *C. parvum* (3), *C. serpentis* (2)
Hallinger, 2020	2015–2019	Germany	CA	571	1	0.2	MIC	*Cryptosporidium* spp. (1)
Zhang, 2020	2019	China	CA	273	17	6.2	MOL	*C. serpentis* (10), *C. varanii* (7)
Chai, 2021	2019	China	CA	609	12	2	MOL	*C. serpentis* (12)
Lobao, 2023	2021–2022	Brazil	CA	189	31	16.4	MOL	*C. tyzzeri* (12), *C. parvum* (14), *Cryptosporidium* spp. (5)
O'Hanlon, 2023	2008–2019	USA	W/F	227	1	0.4	MOL	*C. serpentis* (1)
Mendoza‐Roldan, 2024	2023–2024	Italy	CA	112	4	3.6	MOL	*Cryptosporidium* spp. (4)
Zhang, 2024	2022–2023	China	CA	750	225	30	MOL	*C. serpentis* (149), *C. varanii* (76)
Beck, 2025	UC	USA	W/F	209	22	10.4	MOL	*C. serpentis* (22)
Žahourek, 2025	2021–2022	Czech Republic	CA	131	1	0.8	MOL	*C. tyzzeri* (1)
Pollakova, 2025	UC	Indonesia	CA	40	6	15	MOL	*C. tyzzeri* (2), *C. hominis* (2), *C. parvum* (2)
Louro, 2025	2022–2023	Spain/Portugal	CA	14	7	50	MOL	*C. muris* (2), *C. tyzzeri* (2), *C. serpentis* (3)
Li, 2025a	2018–2020	China	CA	331	81	24.5	MOL	*C. serpentis* (67), *C. tyzzeri* (5), *C. varanii* (4), *C. muris* (3), *C. parvum* (2)
Li, 2025b	2018–2020	China	W/F	26	12	46.2	MOL	*C. serpentis* (10), *C. tyzzeri* (1), *Cryptosporidium* spp. (1)
Sutthikornchai, 2026	2022–2023	Thailand	CA	9	0	0	MOL	—

Abbreviations: CA, captive‐associated; MIC, microscopic detection method; MOL, molecular detection method; UC, unclear; W/F, wild/free‐ranging.

### 3.3 Overall Prevalence of *Cryptosporidium* spp. in Snakes

**FIGURE 2:**
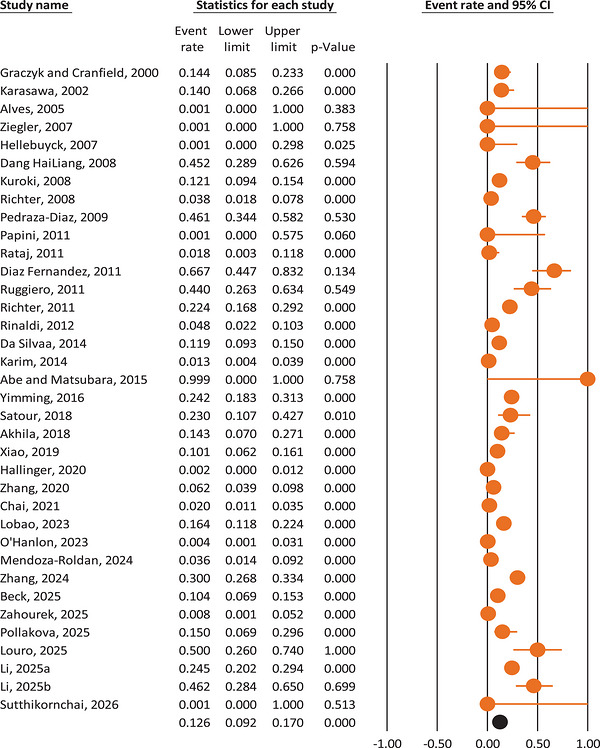
Forest plot showing the pooled prevalence of *Cryptosporidium* spp. in snakes. Orange lines represent 95% CIs for individual study prevalences, with orange circles indicating point estimates. The bottom black circle represents the pooled prevalence and its 95% CI.

The pooled prevalence of *Cryptosporidium* spp. in snakes was estimated at 12.6% (95% CI: 9.2%–17.0%) using a random‐effects model. Considerable between‐study heterogeneity was observed (*Q* = 442.3, *I* ^2^ = 92.1%, *p* < 0.001), indicating substantial variability among the included studies (Figure 2).

### 3.4 Subgroup Analyses

Subgroup analyses according to publication year, continent, country, snake management status, sample size and diagnostic method are summarized in Table 2.

**TABLE 2:** Subgroup analysis of *Cryptosporidium* spp. prevalence in snakes according to publication year, continent, country, snake type, sample size, and diagnostic method.

Subgroup variable	Prevalence % (95% CI)	Heterogeneity (*Q*)	No. studies/datasets	df (*Q*)	*I* ^2^ (%)	*p*‐value
Publication yea**r**						
≤ 2014	14.7 (9–23.2)	167.4	17	16	90.4	< 0.05
> 2014	10.9 (7–16.6)	262.9	19	18	93.2	< 0.05
Continent						
Africa	23 (10.7–42.7)	0	1	0	0	> 0.05
Asia	14.2 (8.9–21.9)	229.5	14	13	94.3	< 0.05
Europe	7.3 (2.7–18)	154.6	13	12	92.2	< 0.05
North America	7.3 (2.8–17.8)	11.8	4	3	74.5	> 0.05
South America	18.3 (11–28.9)	17.8	4	3	83.1	< 0.05
Sample size						
≤ 100	27.7 (18.1–40)	68.7	17	16	76.7	< 0.05
> 100	7.2 (4.8–10.9)	335.9	19	18	94.6	< 0.05
Country						
Austria	9.9 (1.5–43.6)	21.6	2	1	95.4	< 0.05
Belgium	0.1 (0–29.8)	0	1	0	0	> 0.05
Brazil	18.3 (11–28.9)	17.8	4	3	83.1	< 0.05
China	13.2 (6.6–24.6)	201.1	8	7	96.5	< 0.05
Czech Republic	0.8 (0.1–5.2)	0	1	0	0	> 0.05
Egypt	23 (10.7–42.7)	0	1	0	0	> 0.05
Germany	0.2 (0–1.2)	0	1	0	0	> 0.05
India	14.3 (7–27.1)	0	1	0	0	> 0.05
Indonesia	15 (6.9–29.6)	0	1	0	0	> 0.05
Italy	9.3 (0.9–53.1)	47.5	4	3	93.7	< 0.05
Japan	12.1 (9.4–15.4)	0.2	2	1	0	> 0.05
Portugal	0.1 (0–100)	0	1	0	0	> 0.05
Slovenia	1.8 (0.3–11.8)	0	1	0	0	> 0.05
Spain	46.8 (36.1–57.8)	0.1	2	1	0	> 0.05
Thailand	24.2 (18.2–31.3)	0.3	2	1	0	> 0.05
USA	7.3 (2.8–17.8)	11.8	4	3	74.5	> 0.05
Snake type						
Captive‐associated	12.7 (9.2–17.4)	406.7	32	31	92.4	< 0.05
Wild/free‐ranging	8.7 (1.3–40.6)	32.5	4	3	90.8	< 0.05
Diagnostic method						
MIC	8.8 (3.6–20.2)	51.7	9	8	84.5	< 0.05
MOL	13.3 (9.4–18.6)	389.6	27	26	93.3	< 0.05

Based on publication year, studies published before or during 2014 showed a higher pooled prevalence (14.7%; 95% CI: 9.0%–23.2%) compared with studies published after 2014 (10.9%; 95% CI: 7.0%–16.6%) (Figure S1).

Among continents, the highest pooled prevalence was observed in Africa (23%; 95% CI: 10.7%–42.7%), followed by South America (18.3%; 95% CI: 11.0%–28.9%), Asia (14.2%; 95% CI: 8.9%–21.9%), Europe (7.3%; 95% CI: 2.7%–18.0%) and North America (7.3%; 95% CI: 2.8%–17.8%). However, heterogeneity remained substantial in most continental subgroup analyses (Figure S2).

Country‐level analyses demonstrated considerable variation in prevalence estimates. The highest pooled prevalence was observed in Spain (46.8%; 95% CI: 36.1%–57.8%) and Thailand (24.2%; 95% CI: 18.2%–31.3%), whereas very low prevalence estimates were reported in countries such as Belgium, Germany, Portugal and the Czech Republic (Figure S3).

Studies with sample sizes ≤ 100 showed a significantly higher pooled prevalence (27.7%; 95% CI: 18.1%–40.0%) than studies with sample sizes > 100 (7.2%; 95% CI: 4.8%–10.9%) (Figure S4).

Regarding snake management status, captive‐associated snake populations demonstrated a pooled prevalence of 12.7% (95% CI: 9.2%–17.4%), whereas wild/free‐ranging snakes showed a pooled prevalence of 8.7% (95% CI: 1.3%–40.6%). Nevertheless, the number of studies investigating wild populations was limited (Figure S5).

According to diagnostic method, molecular studies reported a higher pooled prevalence (13.3%; 95% CI: 9.4%–18.6%) compared with microscopic studies (8.8%; 95% CI: 3.6%–20.2%). Substantial heterogeneity persisted across nearly all subgroup analyses (Figure S6).

### 3.5 Genetic Diversity of *Cryptosporidium* spp. in Snakes

A total of eight *Cryptosporidium* species were identified among snake populations, including *C. serpentis*, *C. tyzzeri*, *C. varanii*, *C. muris*, *C. parvum*, *C. baileyi*, *C. andersoni* and *C. hominis* (Table 1).

The most frequently reported species were *C. serpentis*, *C. tyzzeri*, *C. varanii* and *C. muris*. All eight identified species were reported from captive‐associated snake populations, whereas only *C. serpentis* and *C. tyzzeri* were detected in wild/free‐ranging snakes.

Several zoonotic or potentially zoonotic species were also identified, including *C. muris*, *C. parvum*, *C. baileyi*, *C. andersoni*, *C. tyzzeri* and *C. hominis*. Notably, most zoonotic species were reported exclusively from captive‐associated snake populations.

In several studies, positive samples were reported only as *Cryptosporidium* spp. without molecular species identification. Additionally, some microscopy‐based studies reported species‐level findings without molecular confirmation.

### 3.6 Sensitivity Analysis

Sensitivity analysis using a leave‐one‐out approach demonstrated that sequential removal of individual studies did not substantially affect the pooled prevalence estimate. The pooled prevalence remained relatively stable, ranging from 11.7% to 13.6%, indicating acceptable robustness of the overall findings (Figure S7).

### 3.7 Meta‐Regression Analysis

**FIGURE 3:**
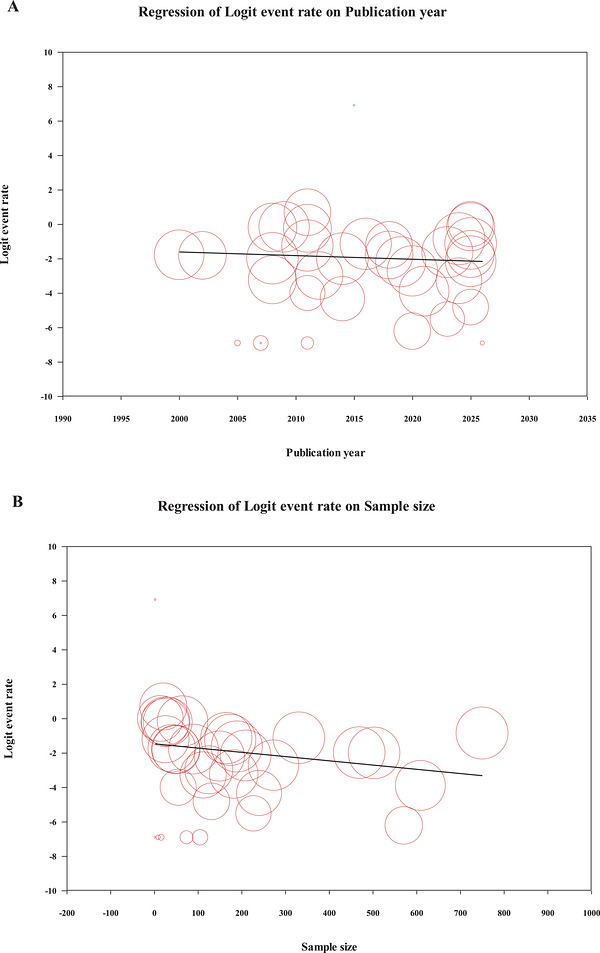
Bubble plots revealing meta‐regression analyses assessing the effect of (A) publication year and (B) sample size on the prevalence of *Cryptosporidium* spp. in snakes. In each plot, the size of the bubbles is proportional to the weight of the study in the meta‐analysis. The fitted regression line represents the relationship between the covariate and the reported prevalence.

Univariable meta‐regression analysis demonstrated that publication year was not significantly associated with variations in *Cryptosporidium* spp. prevalence among snakes (coefficient = −0.0213, *p* = 0.4020). In contrast, sample size showed a significant negative association with prevalence estimates (coefficient = −0.0025, *p* = 0.0128), indicating that smaller studies tended to report higher prevalence rates. Despite this significant association, a considerable proportion of heterogeneity remained unexplained (Figure 3).

### 3.8 Publication Bias

**FIGURE 4:**
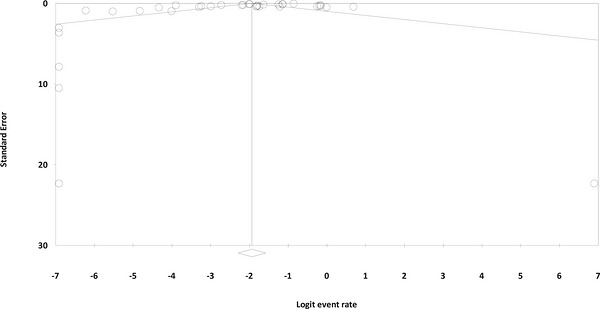
Funnel plot revealing publication bias in the current systematic review and meta‐analysis. The *x*‐axis represents the logit event rate, while the *y*‐axis displays the standard error.

Visual inspection of the funnel plot suggested potential asymmetry among included studies. Egger's regression test further indicated significant publication bias (*p* = 0.02) (Figure 4).

## 4 Discussion

*Cryptosporidium* spp. are important protozoan parasites infecting a wide range of vertebrate hosts, including reptiles (Brener et al. 2022). Although cryptosporidiosis in snakes has been recognized for decades, information regarding its global prevalence, molecular diversity, and zoonotic significance has remained fragmented. Therefore, the present systematic review and meta‐analysis was conducted to comprehensively synthesize available epidemiological and molecular evidence concerning *Cryptosporidium* spp. in snakes worldwide.

The pooled prevalence of *Cryptosporidium* spp. in snakes observed in the present study (12.6%) was generally comparable to prevalence estimates reported in several previous systematic reviews and meta‐analyses conducted in other animal hosts. Relatively similar pooled prevalence rates have been reported in camels (13.8%) (Mahdavi et al. 2024) and pigeons (10%) (Mahdavi et al. 2025), whereas lower weighted prevalence estimates were documented in dogs (6%–8%) (Taghipour et al. 2020), cats (6%) (Taghipour et al. 2021), rabbits (9%) (Ghorbani et al. 2025) and *Equus* animals (7.6%) (X. Li et al. 2022). In contrast, substantially higher pooled prevalence rates have been reported in pigs (16.3%) (Chen et al. 2023) and particularly in cattle (Buchanan et al. 2025), where prevalence in calves and pre‐weaned animals frequently exceeded 27%–37.5%. These differences likely reflect variations in host susceptibility, management systems, environmental exposure, husbandry conditions, geographic distribution, age structure and diagnostic methodologies among animal populations.

The high heterogeneity observed in the present meta‐analysis likely reflects the marked epidemiological and methodological diversity among the included studies (Asghari et al. 2026). Snake species, geographic origin, captive management conditions, diagnostic approaches, study design and sample size differed substantially across investigations. Furthermore, many studies involved relatively small sample sizes or opportunistic sampling from captive collections, which may have increased variability and reduced representativeness.

Subgroup analysis according to publication year showed only modest differences in pooled prevalence estimates between studies published in or before 2014 and those published after 2014. The year 2014 was not selected because of a specific epidemiological event or diagnostic milestone, but rather to provide two subgroups of nearly equal size, thereby improving the statistical balance of subgroup comparisons. Since sampling year would more accurately reflect temporal changes in infection prevalence, it would have been preferable to use this variable for temporal analyses. However, sampling periods were inconsistently reported across the included studies, precluding a reliable subgroup analysis based on the actual time of sample collection. Consequently, publication year was used as a pragmatic surrogate and the corresponding findings should be interpreted cautiously. Consistent with this observation, the meta‐regression analysis demonstrated that publication year was not significantly associated with the reported prevalence of *Cryptosporidium* spp., suggesting that no clear temporal trend could be identified from the currently available published evidence.

The significantly higher prevalence observed in studies with smaller sample sizes suggests the possibility of small‐study effects and potential overestimation of prevalence in limited datasets. This interpretation is additionally supported by the significant publication bias detected through Egger's regression test. Smaller studies may preferentially originate from outbreak investigations, clinically affected collections, or facilities with known infection problems, thereby increasing the probability of reporting positive findings. Moreover, differences in diagnostic methodology may also have contributed to funnel plot asymmetry. Molecular techniques generally provide greater analytical sensitivity than conventional microscopic methods, particularly for detecting low‐intensity infections and accurately differentiating *Cryptosporidium* species. Consequently, studies employing molecular diagnostics may have been more likely to report positive findings than microscopy‐based investigations. Additional contributors to publication bias may include the preferential publication of studies reporting positive results, underreporting of negative surveys and the limited availability of epidemiological data from many geographic regions. Therefore, both the pooled prevalence estimate and the observed publication bias should be interpreted cautiously in light of these methodological and reporting differences.

The significant association identified between sample size and prevalence in the meta‐regression further supports the influence of study size on the reported epidemiological estimates. Smaller studies tended to report higher prevalence values than larger investigations, which may reflect selective sampling of clinically affected collections, localized outbreaks or populations with known infection status. In contrast, larger epidemiological surveys generally include more heterogeneous populations and may therefore provide more conservative prevalence estimates. Nevertheless, sample size alone is unlikely to explain the substantial heterogeneity observed in the present meta‐analysis, indicating that additional biological and methodological factors also contribute to the variability among studies.

Captive‐associated snakes demonstrated a higher pooled prevalence than wild/free‐ranging snakes, although the number of available studies on wild populations was limited. This finding is biologically plausible because captivity‐related factors, including high animal density, chronic stress, repeated environmental contamination, shared husbandry equipment and close confinement, may facilitate parasite transmission and persistence. In contrast, transmission dynamics in wild snake populations are likely influenced by lower host density, greater environmental dispersion and reduced direct contact between individuals. Nevertheless, the wide CI observed for wild snake populations highlights the limited available evidence and emphasizes the need for further surveillance studies in free‐ranging snakes.

Considerable variation in the reported prevalence of *Cryptosporidium* spp. was observed across different countries and continents. However, these findings should be interpreted with substantial caution because the available epidemiological evidence remains highly limited and unevenly distributed geographically. Most countries and several continental subgroup estimates were represented by only one to four/five studies, which considerably limits the reliability and representativeness of pooled prevalence estimates. China contributed the highest number of studies included in the present systematic review (*n* = 8), whereas many other countries were represented by only a single dataset. Consequently, the reported prevalence estimates cannot be considered fully representative of the actual national or continental epidemiological situation of *Cryptosporidium* spp. in snake populations. Moreover, differences in snake species composition, captive management practices, environmental conditions, sampling strategies, diagnostic methodologies and study quality may have further influenced the observed geographical variability. Importantly, the currently available evidence is insufficient to accurately estimate the true global prevalence of *Cryptosporidium* spp. in snakes, and the pooled estimates presented in the present meta‐analysis primarily reflect the limited published data currently available rather than the complete worldwide epidemiological distribution of this parasite. Therefore, more extensive, geographically representative and standardized molecular epidemiological investigations are required to better clarify the regional and global epidemiology of *Cryptosporidium* spp. in snake populations.

Molecular studies yielded higher prevalence estimates than microscopy‐based investigations, which is consistent with the greater sensitivity and specificity of molecular diagnostic methods. Microscopic identification of *Cryptosporidium* spp. oocysts in reptiles can be challenging because oocyst morphology overlaps among species and low‐level shedding may escape microscopic detection. Moreover, species‐level identification based solely on morphology may lead to diagnostic inaccuracies. The increasing use of PCR‐based methods has considerably improved understanding of reptile‐associated *Cryptosporidium* spp. diversity and transmission dynamics.

Considerable diversity of *Cryptosporidium* species has been reported across animal hosts worldwide, reflecting the complex epidemiology and broad host adaptability of this protozoan parasite. In the present study, eight *Cryptosporidium* species were identified in snakes, including *C. serpentis*, *C. tyzzeri*, *C. varanii*, *C. muris*, *C. parvum*, *C. baileyi*, *C. andersoni* and *C. hominis*. Among these, *C. serpentis*, *C. tyzzeri*, *C. varanii* and *C. muris* were the most frequently reported species. Various species diversity has been reported in animals such as camels (Mahdavi et al. 2024), pigs (Chen et al. 2023), pigeons (Mahdavi et al. 2025), cattle (Buchanan et al. 2025), rabbits (Ghorbani et al. 2025), dogs (Taghipour et al. 2020) and cats (Taghipour et al. 2021), although the dominant species differed considerably among hosts. For example, *C. parvum* predominated in cattle, *C. baileyi* and *C. meleagridis* in pigeons, *C. scrofarum* and *C. suis* in pigs, *C. canis* in dogs, *C. felis* in cats, *C. cuniculus* in rabbits and *C. parvum* together with *C. andersoni* in camels. In contrast, *C. serpentis* remained the predominant and most host‐adapted species detected in snakes, consistent with its recognized pathogenic role in reptilian cryptosporidiosis. However, it should be noted that species distribution data were synthesized descriptively rather than through quantitative species‐specific meta‐analysis due to limited molecular reporting across studies. Therefore, the observed frequency of certain species may partly reflect differences in study availability, molecular characterization practices, and reporting frequency among investigations.

Importantly, several zoonotic or potentially zoonotic species were also identified in snakes, including *C. tyzzeri*, *C. parvum*, *C. muris*, *C. andersoni*, *C. baileyi* and *C. hominis* (Ryan et al. 2021, Shi et al. 2025), highlighting the potential One Health relevance of snake‐associated cryptosporidiosis. Among these, *C. tyzzeri* was particularly noteworthy because it was reported from both captive‐associated and wild/free‐ranging snakes. This finding may suggest broader environmental circulation and possible interspecies transmission pathways for this species. Since *C. tyzzeri* has been reported from humans and a broad range of mammalian hosts (Ryan et al. 2021, Wagnerová et al. 2015), its detection in both captive‐associated and wild/free‐ranging snakes may indicate wider environmental dissemination and complex interspecies transmission dynamics involving multiple host species. Nevertheless, the ecological significance of this finding remains uncertain, and currently available evidence is insufficient to determine whether snakes function as true biological hosts, transient carriers or incidental reservoirs of this species.

The detection of additional zoonotic species such as *C. parvum* and *C. hominis* further emphasizes the potential public health importance of these findings. However, interpretation of zoonotic *Cryptosporidium* species detected in snakes should be approached cautiously. Because snakes are carnivorous reptiles commonly fed rodents, birds or other prey animals, some molecular detections may represent transient passage of ingested oocysts or pseudoparasitism rather than active infection or sustained host adaptation. This possibility is particularly relevant for mammalian‐ and avian‐associated species such as *C. muris*, *C. parvum*, *C. andersoni* and *C. baileyi* (Ryan et al. 2021, Louro et al. 2025). Similarly, the presence of *C. hominis*, which is predominantly considered a human‐associated species (Ryan et al. 2021), may reflect environmental contamination, reverse zoonotic transmission, or incidental carriage. Furthermore, several included studies reported positive samples only as *Cryptosporidium* spp. without molecular species identification, whereas some microscopy‐based investigations assigned species‐level identifications without molecular confirmation. Consequently, the currently recognized diversity of *Cryptosporidium* spp. in snakes may be either underestimated due to incomplete molecular characterization or overestimated because of potential diagnostic misclassification. These findings collectively highlight the need for further standardized molecular epidemiological investigations to better clarify the host specificity, transmission ecology, and zoonotic significance of *Cryptosporidium* spp. in snake populations.

The present study has several strengths. To our knowledge, this is the first systematic review and meta‐analysis specifically evaluating the global prevalence, genetic diversity and zoonotic implications of *Cryptosporidium* spp. in snakes. The study incorporated a broad geographic range, multiple diagnostic methods, subgroup analyses, sensitivity analyses, meta‐regression and molecular diversity assessments. However, several limitations should also be acknowledged. The number of studies investigating wild snake populations was limited, substantial heterogeneity was present, publication bias was detected, and many included studies had relatively small sample sizes. Additionally, uneven geographic distribution of studies and inconsistent molecular characterization limited the precision of some subgroup analyses and species‐level interpretations.

## 5 Conclusion

The present systematic review and meta‐analysis demonstrated that *Cryptosporidium* infection is relatively common in snake populations worldwide, particularly among captive‐associated snakes. Considerable genetic diversity was identified, including both snake‐adapted and potentially zoonotic species. *C. serpentis* remained the predominant species detected in snakes; however, the occurrence of zoonotic species such as *C. tyzzeri*, *C. parvum*, *C. muris*, *C. andersoni*, *C. baileyi* and *C. hominis* highlights the potential epidemiological importance of snake‐associated cryptosporidiosis within a One Health context. Despite these findings, the current evidence base remains limited by substantial heterogeneity, uneven geographical representation and incomplete molecular characterization. Furthermore, the detection of zoonotic species in snakes does not necessarily confirm active infection or reservoir competence. Future studies employing standardized molecular approaches, larger sample sizes and expanded surveillance of wild snake populations are required to better clarify the ecology, transmission dynamics, host specificity and public health significance of *Cryptosporidium* spp. in snakes.

## Author Contributions

**Ali Asghari** and **Farajolah Maleki** planned and designed the study. **Ali Pouryousef**, **Kambiz Karimi**, **Amin Kheiri** and **Mohammad Reza Soltani** were involved in the methodology and data extraction. Ali Asghari and Farajolah Maleki conducted the statistical analysis. Ali Asghari, Farajolah Maleki, and Ali Pouryousef wrote the manuscript and revised it. All authors have read and approved the final manuscript.

## Funding

The authors have nothing to report.

## Ethics Statement

This study was conducted exclusively using data extracted from previously published articles and publicly available scientific sources. No human participants, animals, or biological specimens were directly involved in the present investigation. The study protocol, including the use of secondary data, was reviewed and approved by the Ethics Committee of Khoy University of Medical Sciences, Khoy, Iran (approval no. IR.KHOY.REC.1404.040).

## Conflicts of Interest

The authors declare no conflicts of interest.

## Supporting information




**Supplementary Fig 1**: The pooled prevalence of *Cryptosporidium* spp. in snakes based on publication year. Orange indicates the prevalence from each study, while black shows the weighted prevalence.

**Supplementary Fig 2**: The pooled prevalence of *Cryptosporidium* spp. in snakes based on continent. Orange indicates the prevalence from each study, while black shows the weighted prevalence.

**Supplementary Fig 3**: The pooled prevalence of *Cryptosporidium* spp. in snakes based on country. Orange indicates the prevalence from each study, while black shows the weighted prevalence.

**Supplementary Fig 4**: The pooled prevalence of *Cryptosporidium* spp. in snakes based on sample size. Orange indicates the prevalence from each study, while black shows the weighted prevalence.

**Supplementary Fig 5**: The pooled prevalence of *Cryptosporidium* spp. in snakes based on snake management status (snake type). Orange indicates the prevalence from each study, while black shows the weighted prevalence.

**Supplementary Fig 6**: The pooled prevalence of *Cryptosporidium* spp. in snakes based on the diagnostic method. Orange indicates the prevalence from each study, while black shows the weighted prevalence.

**Supplementary Fig 7**: Sensitivity analysis assessing the influence of individual studies on pooled estimates.

**Supporting Information**: PRISMA 2020 Checklist

**Supplementary Table 2**: JBI critical appraisal checklist applied for included studies.

## Data Availability

The data that supports the findings of this study are available in the Supporting Information of this article.

## References

[vms371167-bib-0001] Abe, N. , and K. Matsubara . 2015. “Molecular Identification of *Cryptosporidium* Isolates From Exotic Pet Animals in Japan.” Veterinary Parasitology 209: 254–257. 25801359 10.1016/j.vetpar.2015.02.035

[vms371167-bib-0002] Adeyemo, F. E. , G. Singh , P. Reddy , F. Bux , and T. A. Stenström . 2019. “Efficiency of Chlorine and UV in the Inactivation of *Cryptosporidium* and *Giardia* in Wastewater.” PLoS ONE 14: e0216040. 31083664 10.1371/journal.pone.0216040 PMC6513095

[vms371167-bib-0003] Akhila, S. , V. S. Sukanya , A. Anto , and S. Karunakaran . 2018. “Prevalence of Endoparasites in Captive Snakes of Kerala, India.” Annals of Parasitology 64: 129–135. 29983025 10.17420/ap6402.144

[vms371167-bib-0004] Alves, M. , L. Xiao , V. Lemos , et al. 2005. “Occurrence and Molecular Characterization of *Cryptosporidium* spp. in Mammals and Reptiles at the Lisbon Zoo.” Parasitology Research 97: 108–112. 15986253 10.1007/s00436-005-1384-9

[vms371167-bib-0005] Asghari, A. , M. R. Mohammadi , R. Norouzi , et al. 2026. “A Global Overview of Microsporidia Infection in HIV/AIDS Patients: An Updated Systematic Review and Meta‐Analysis.” International Health ihaf163. 41601338 10.1093/inthealth/ihaf163 PMC13530294

[vms371167-bib-0006] Beck, G. M. 2025. A Cryptic Concern for Snakes of Killdeer Plains Wildlife Area: Cryptosporidium serpentis . Ohio Northern University.

[vms371167-bib-0007] Brener, B. , E. Burgarelli , M. Suarez , and L. Keidel . 2022. “Cryptosporidiosis in Reptiles From Brazil: An Update for Veterinary Medicine.” Parasitologia 2: 228–236.

[vms371167-bib-0008] Buchanan, R. , P. Wieckowski , E. Matechou , F. Katzer , A. D. Tsaousis , and M. Farré . 2025. “Global Prevalence of *Cryptosporidium* Infections in Cattle: A Meta‐Analysis.” Current Research in Parasitology & Vector‐Borne Diseases 7: 100264. 40487329 10.1016/j.crpvbd.2025.100264 PMC12144461

[vms371167-bib-0009] Certad, G. , E. Viscogliosi , M. Chabé , and S. M. Cacciò . 2017. “Pathogenic Mechanisms of *Cryptosporidium* and *Giardia* .” Trends in Parasitology 33: 561–576. 28336217 10.1016/j.pt.2017.02.006

[vms371167-bib-0010] Chai, Y. , H. Liu , L. Deng , et al. 2021. “Prevalence and Molecular Characterization of *Cryptosporidium serpentis* in Captive Snakes in China.” Journal of Parasitology 107: 163–171. 33662118 10.1645/20-100

[vms371167-bib-0011] Chen, X. , Q.‐Y. Zhao , Y.‐M. Chen , H. Tariq , and L.‐S. Zang . 2024. “Ontogenetic Morphological Changes of the Venom Apparatus in 4 Eupelmid Egg Parasitoids.” Parasitology 151: 185–190. 38186337 10.1017/S0031182023001282 PMC10941032

[vms371167-bib-0012] Dang, H. L. , G. S. He , and L. X. Zhang . 2008. “Investigation on the Prevalence of *Cryptosporidium* in Reptiles and Amphibians in Shanghai.” Chinese Journal of Zoonoses 24: 179.

[vms371167-bib-0013] da Silva, D. C. , P. Paiva , A. A. Nakamura , et al. 2014. “The Detection of *Cryptosporidium serpentis* in Snake Fecal Samples by Real‐Time PCR.” Veterinary Parasitology 204: 134–138. 24880646 10.1016/j.vetpar.2014.05.012

[vms371167-bib-0014] Diaz Fernandez, P. D. , S. Rota , B. Marchesi , et al. 2011. “Molecular Identification of *Cryptosporidium* Isolates From Captive Snakes in Italy.” In XII Congreso iberico Parasitologia. Sociedad Espanola de Parasitologia.

[vms371167-bib-0015] Garcia‐R, J. C. , A. B. Pita , N. Velathanthiri , A. Pas , and D. T. S. Hayman . 2023. “Mammal‐Related *Cryptosporidium* Infections in Endemic Reptiles of New Zealand.” Parasitology Research 122: 1239–1244. 36959486 10.1007/s00436-023-07824-4 PMC10097775

[vms371167-bib-0016] Ghorbani, A. , A. Asghari , M. R. Mohammadi , et al. 2025. “ *Cryptosporidium* in Rabbits: A Global Systematic Review and Meta‐Analysis of Prevalence, Species/Genotypes Distribution and Zoonotic Significance.” Veterinary Medicine and Science 11: e70309. 40103565 10.1002/vms3.70309 PMC11920741

[vms371167-bib-0017] Graczyk, T. K. , and M. R. Cranfield . 2000. “ *Cryptosporidium serpentis* Oocysts and Microsporidian Spores in Feces of Captive Snakes.” Journal of Parasitology 86: 413–414. 10780564 10.1645/0022-3395(2000)086[0413:CSOAMS]2.0.CO;2

[vms371167-bib-0018] Hallinger, M. J. , A. Taubert , and C. Hermosilla . 2020. “Occurrence of *Kalicephalus*, *Strongyloides*, and *Rhabdias* Nematodes as Most Common Gastrointestinal Parasites in Captive Snakes of German Households and Zoological Gardens.” Parasitology Research 119: 947–956. 31950252 10.1007/s00436-019-06526-0

[vms371167-bib-0019] Hellebuyck, T. , D. Vancraeynest , T. Geurden , E. Claerebout , and F. Pasmans . 2007. “Prevalence of Cryptosporidiosis in Belgian Snake Collections.” Vlaams Diergeneeskd Tijdschr 76.

[vms371167-bib-0020] Helmy, Y. A. , and H. M. Hafez . 2022. “Cryptosporidiosis: From Prevention to Treatment, a Narrative Review.” Microorganisms 10: 2456. 36557709 10.3390/microorganisms10122456 PMC9782356

[vms371167-bib-0021] Karasawa, A. S. M. , R. J. Silva , L. M. Mascarini , T. H. Barrella , and C. A. M. Lopes . 2002. “Occurrence of *Cryptosporidium* (Apicomplexa, Cryptosporidiidae) in *Crotalus durissus terrificus* (Serpentes, Viperidae) in Brazil.” MemÃ3rias do Instituto Oswaldo Cruz 97: 779–781. 10.1590/s0074-02762002000600004 12386695

[vms371167-bib-0022] Karim, M. R. , F. Yu , J. Li , et al. 2014. “First Molecular Characterization of Enteric Protozoa and the Human Pathogenic Microsporidian, *Enterocytozoon bieneusi*, in Captive Snakes in China.” Parasitology Research 113: 3041–3048. 24906991 10.1007/s00436-014-3967-9

[vms371167-bib-0023] Kuroki, T. , S. Izumiyama , K. Yagita , et al. 2008. “Occurrence of *Cryptosporidium* sp. in Snakes in Japan.” Parasitology Research 103: 801–805. 18548279 10.1007/s00436-008-1045-x

[vms371167-bib-0024] Li, F. , X. Wang , L. Xiao , Y. Feng , and Y. Guo . 2025. “High Prevalence and Pathogenicity of *Cryptosporidium serpentis* in Snakes in Southern China.” Current Research in Parasitology & Vector‐Borne Diseases 8: 100287. 40677561 10.1016/j.crpvbd.2025.100287 PMC12269443

[vms371167-bib-0025] Li, X.‐M. , H.‐L. Geng , Y.‐J. Wei , et al. 2022. “Global Prevalence and Risk Factors of *Cryptosporidium* Infection in *Equus*: A Systematic Review and Meta‐Analysis.” Frontiers in Cellular and Infection Microbiology 12: 1072385. 36506009 10.3389/fcimb.2022.1072385 PMC9732577

[vms371167-bib-0026] Lobão, L. F. , L. L. Corrêa , S. F. Bruno , S. da Silva , C. M. A. Uchôa , and A. da Silva Barbosa . 2023. “Diagnosis of Endoparasite Species and Subtypes of *Cryptosporidium* spp. With One Health Importance, in Feces From Captive Snakes in Rio de Janeiro, Brazil.” Parasitology International 97: 102797. 37604363 10.1016/j.parint.2023.102797

[vms371167-bib-0027] Louro, M. , L. Hernandez , J. Antunes , L. Madeira de Carvalho , I. Pereira da Fonseca , and J. Gomes . 2025. “ *Cryptosporidium* spp. in Reptiles: Detection Challenges, Molecular Characterization and Zoonotic Risk.” Food and Waterborne Parasitology 40: e00272. 40546388 10.1016/j.fawpar.2025.e00272 PMC12182340

[vms371167-bib-0028] Mahdavi, F. , F. Maleki , M. R. Mohammadi , A. Asghari , and B. Mohammadi‐Ghalehbin . 2024. “Global Epidemiology and Species/Genotype Distribution of *Cryptosporidium* in Camels: A Systematic Review and Meta‐Analysis.” Food and Waterborne Parasitology 36: e00235. 39109171 10.1016/j.fawpar.2024.e00235 PMC11298603

[vms371167-bib-0029] Mahdavi, F. , M. Mamizadeh , M. R. Mohammadi , et al. 2026. “Global Prevalence and Genetic Diversity of *Cryptosporidium* spp. in Pigeons: A Systematic Review and Meta‐Analysis.” Comparative Immunology, Microbiology and Infectious Diseases 126: 102437. 41468669 10.1016/j.cimid.2025.102437

[vms371167-bib-0030] Mahmudunnabi, R. G. , S. Kasetsirikul , N. Soda , et al. 2024. “Critical Evaluation of Current Isolation, Detection, and Genotyping Methods of *Cryptosporidium* Species and Future Direction.” Environmental Science: Water Research & Technology 10: 1527–1551.

[vms371167-bib-0031] Mamizadeh, M. , F. Maleki , M. R. Mohammadi , L. Shamsi , A. Asghari , and A. Pouryousef . 2025. “Seroprevalence and Risk Factors for *Toxoplasma gondii* Infection in Solid Organ Transplant Patients: A Global Systematic Review and Meta‐Analysis.” Parasite Epidemiology and Control 29: e00421. 40129460 10.1016/j.parepi.2025.e00421 PMC11932682

[vms371167-bib-0032] Mendoza‐Roldan, J. A. , D. Modry , and D. Otranto . 2020. “Zoonotic Parasites of Reptiles: A Crawling Threat.” Trends in Parasitology 36: 677–687. 32448703 10.1016/j.pt.2020.04.014 PMC7203055

[vms371167-bib-0033] Mendoza‐Roldan, J. A. , L. Perles , E. Filippi , et al. 2024. “Parasites and Microorganisms Associated With the Snakes Collected for the “Festa Dei Serpari” in Cocullo, Italy.” PLOS Neglected Tropical Diseases 18: e0011973. 38381797 10.1371/journal.pntd.0011973 PMC10911609

[vms371167-bib-0034] Moher, D. , L. Shamseer , M. Clarke , et al. 2015. “Preferred Reporting Items for Systematic Review and Meta‐Analysis Protocols (PRISMA‐P) 2015 Statement.” Systematic Reviews 4: 1–9. 25554246 10.1186/2046-4053-4-1 PMC4320440

[vms371167-bib-0035] O'Hanlon, B. M. , J. E. Bogan , J. C. Godwin , et al. 2023. “ *Cryptosporidium serpentis* Surveillance in Free‐Ranging Snakes to Inform a Reintroduction Strategy for the Eastern Indigo Snake (*Drymarchon couperi*).” Journal of Wildlife Diseases 59: 176–180. 36584345 10.7589/JWD-D-22-00055

[vms371167-bib-0036] Page, M. J. , J. E. McKenzie , P. M. Bossuyt , et al. 2021. “The PRISMA 2020 Statement: An Updated Guideline for Reporting Systematic Reviews.” BMJ 372: n71. 33782057 10.1136/bmj.n71 PMC8005924

[vms371167-bib-0037] Papini, R. , C. Manetti , and F. Mancianti . 2011. “Coprological Survey in Pet Reptiles in Italy.” Veterinary Record 169: 207. 21795307 10.1136/vr.d4398

[vms371167-bib-0038] Pedraza‐Díaz, S. , L. M. Ortega‐Mora , B. A. Carrión , V. Navarro , and M. Gómez‐Bautista . 2009. “Molecular Characterisation of *Cryptosporidium* Isolates From Pet Reptiles.” Veterinary Parasitology 160: 204–210. 19101086 10.1016/j.vetpar.2008.11.003

[vms371167-bib-0039] Polláková, M. , M. Sučik , and V. Petrilla . 2025. “Molecular Detection of Different Species of *Cryptosporidium* in Snakes From Surinam and Indonesia.” Animals 15: 1556. 40509022 10.3390/ani15111556 PMC12153664

[vms371167-bib-0040] Pumipuntu, N. , and S. Piratae . 2018. “Cryptosporidiosis: A Zoonotic Disease Concern.” Veterinary World 11: 681–686. 29915508 10.14202/vetworld.2018.681-686 PMC5993756

[vms371167-bib-0041] Putignani, L. , and D. Menichella . 2010. “Global Distribution, Public Health and Clinical Impact of the Protozoan Pathogen *Cryptosporidium* .” Interdisciplinary Perspectives on Infectious Diseases 2010: 1–39. 10.1155/2010/753512 PMC2913630 20706669

[vms371167-bib-0042] Rataj, A. V. , R. Lindtner‐Knific , K. Vlahović , U. Mavri , and A. Dovč . 2011. “Parasites in Pet Reptiles.” Acta Veterinaria Scandinavica 53: 33. 21624124 10.1186/1751-0147-53-33 PMC3118381

[vms371167-bib-0043] Richter, B. , A. Kübber‐Heiss , H. Weissenböck , and P. Schmidt . 2008. “Detection of *Cryptosporidium* spp., *Entamoeba* spp. and *Monocercomonas* spp. in the Gastrointestinal Tract of Snakes by In‐Situ Hybridization.” Journal of Comparative Pathology 138: 63–71. 18295780 10.1016/j.jcpa.2007.11.001

[vms371167-bib-0044] Richter, B. , N. Nedorost , A. Maderner , and H. Weissenböck . 2011. “Detection of *Cryptosporidium* Species in Feces or Gastric Contents From Snakes and Lizards as Determined by Polymerase Chain Reaction Analysis and Partial Sequencing of the 18S Ribosomal RNA Gene.” Journal of Veterinary Diagnostic Investigation 23: 430–435. 21908271 10.1177/1040638711403415

[vms371167-bib-0045] Rinaldi, L. , M. Capasso , A. D. Mihalca , R. Cirillo , G. Cringoli , and S. Cacciò . 2012. “Prevalence and Molecular Identification of *Cryptosporidium* Isolates From Pet Lizards and Snakes in Italy.” Parasite 19: 437–440. 23193530 10.1051/parasite/2012194437 PMC3671454

[vms371167-bib-0046] Ruggiero, P. C. , R. L. Zacariotti , E. F. Bondan , and M. A. Lallo . 2011. “Prevalência de Cryptosporidium Serpentis em Serpentes de Cativeiro.” Ciência Rural 41: 1975–1978.

[vms371167-bib-0047] Ryan, U. , A. Zahedi , Y. Feng , and L. Xiao . 2021. “An Update on Zoonotic Cryptosporidium Species and Genotypes in Humans.” Animals 11: 3307. 34828043 10.3390/ani11113307 PMC8614385

[vms371167-bib-0048] Satour, N. S. , and A. W. Dewir . 2018. “Some Internal Parasites of Reptiles in Alexandria Province.” Egyptian Veterinary Medical Society of Parasitology Journal 14: 98–114.

[vms371167-bib-0049] Shamsi, L. , M. Mamizadeh , M. R. Mohammadi , et al. 2025. “Prevalence of Free‐Living Amoebae in Various Water Sources in Iran: A Systematic Review and Meta‐Analysis.” Acta Parasitologica 70: 220. 41238952 10.1007/s11686-025-01154-4

[vms371167-bib-0050] Shi, R. , W. Wei , D. Jiao , et al. 2025. “Prevalence and Risk Factors of *Cryptosporidium* in Humans in China: A Systematic Review and Meta‐Analysis.” Microbial Pathogenesis 211: 108255. 41429363 10.1016/j.micpath.2025.108255

[vms371167-bib-0051] Sutthikornchai, C. , A.‐R. Pintong , R. Udonsom , et al. 2026. “Detection of *Cryptosporidium* and *Giardia duodenalis* in Reptiles in Thailand.” Veterinary Medicine and Science 12: e70704. 41317138 10.1002/vms3.70704 PMC12664283

[vms371167-bib-0052] Taghipour, A. , S. Khazaei , S. Ghodsian , et al. 2021. “Global Prevalence of *Cryptosporidium* spp. in Cats: A Systematic Review and Meta‐Analysis.” Research in Veterinary Science 137: 77–85. 33933711 10.1016/j.rvsc.2021.04.015

[vms371167-bib-0053] Taghipour, A. , M. Olfatifar , S. Bahadory , et al. 2020. “The Global Prevalence of *Cryptosporidium* Infection in Dogs: A Systematic Review and Meta‐Analysis.” Veterinary Parasitology 281: 109093. 32278149 10.1016/j.vetpar.2020.109093

[vms371167-bib-0054] Tipu, J. H. , A. Sivertsen , J.‐E. Afset , et al. 2024. “Cryptosporidium Species and Subtypes in Norway: Predominance of *C. parvum* and Emergence of *C. mortiferum* .” Emerging Microbes & Infections 13: 2412624. 39361548 10.1080/22221751.2024.2412624 PMC11485689

[vms371167-bib-0055] Wagnerová, P. , B. Sak , J. McEvoy , et al. 2015. “Genetic Diversity of *Cryptosporidium* spp. Including Novel Identification of the *Cryptosporidium muris* and *Cryptosporidium tyzzeri* in Horses in the Czech Republic and Poland.” Parasitology Research 114: 1619–1624. 25722018 10.1007/s00436-015-4353-y

[vms371167-bib-0056] Xiao, L. , U. M. Ryan , T. K. Graczyk , et al. 2004. “Genetic Diversity of *Cryptosporidium* spp. in Captive Reptiles.” Applied and Environmental Microbiology 70: 891–899. 14766569 10.1128/AEM.70.2.891-899.2004 PMC348785

[vms371167-bib-0057] Xiao, X. , R. Qi , H.‐J. Han , et al. 2019. “Molecular Identification and Phylogenetic Analysis of *Cryptosporidium*, *Hepatozoon* and *Spirometra* in Snakes From Central China.” International Journal for Parasitology: Parasites and Wildlife 10: 274–280. 31700790 10.1016/j.ijppaw.2019.10.001 PMC6829678

[vms371167-bib-0058] Xu, N. , H. Liu , Y. Jiang , et al. 2020. “First Report of *Cryptosporidium viatorum* and *Cryptosporidium occultus* in Humans in China, and of the Unique Novel *C. viatorum* Subtype XVaA3h.” BMC Infectious Diseases 20: 16. 31910816 10.1186/s12879-019-4693-9 PMC6947842

[vms371167-bib-0059] Yimming, B. , K. Pattanatanang , P. Sanyathitiseree , et al. 2016. “Molecular Identification of *Cryptosporidium* Species From Pet Snakes in Thailand.” Korean Journal of Parasitology 54: 423–429. 27658593 10.3347/kjp.2016.54.4.423 PMC5040075

[vms371167-bib-0060] Žahourek, J. , and J. Kvičerová . 2025. “Gastrointestinal Apicomplexan Parasites of Captive Reptiles: Diversity, Causes, Consequences.” BMC Veterinary Research 21: 1–12. 41102773 10.1186/s12917-025-05029-8 PMC12532844

[vms371167-bib-0061] Zhang, H. , Z. Lin , Y. Jiang , et al. 2020. “Molecular Identification of *Cryptosporidium* spp. in Pet Snakes in Beijing, China.” Parasitology Research 119: 3119–3123. 32743725 10.1007/s00436-020-06838-6

[vms371167-bib-0062] Zhang, Y. , Z. Lu , L. He , et al. 2024. “ *Cryptosporidium* spp. in Captive Snakes From 26 Provinces in China: Prevalence, Molecular Characterization, and Symptoms.” Parasite 31: 47. 39109984 10.1051/parasite/2024047 PMC11305116

[vms371167-bib-0063] Ziegler, P. E. , S. E. Wade , S. L. Schaaf , D. A. Stern , C. A. Nadareski , and H. O. Mohammed . 2007. “Prevalence of *Cryptosporidium* Species in Wildlife Populations Within a Watershed Landscape in Southeastern New York State.” Veterinary Parasitology 147: 176–184. 17466459 10.1016/j.vetpar.2007.03.024

