# Supplementary material for: Prevalence, Genetic Diversity, and Zoonotic Transmission Potential of Cryptosporidium spp. in Snakes: A Systematic Review and Meta‐Analysis
Source: Vet Med Sci. 2026 Aug 12;12(5):e71167. doi: 10.1002/vms3.71167 (PMC13465780; doi:10.1002/vms3.71167)
Supplement: Supplementary file 4 [file VMS3-12-e71167-s004.docx]

**Supplementary Fig. 7.** Sensitivity analysis assessing the influence of individual studies on pooled estimates.
